# Cancers associated with Kaposi's sarcoma (KS) in AIDS: a link between KS herpesvirus and immunoblastic lymphoma

**DOI:** 10.1054/bjoc.2001.2052

**Published:** 2001-09-01

**Authors:** E A Engels, P S Rosenberg, M Frisch, J J Goedert

**Affiliations:** Viral Epidemiology Branch and Biostatistics Branch, Division of Cancer Epidemiology and Genetics, National Cancer Institute, 6120 Executive Blvd., Rockville, MD, USA; Biostatistics Branch, Division of Cancer Epidemiology and Genetics, National Cancer Institute, 6120 Executive Blvd., Rockville, MD, USA; Department of Epidemiology Research, Danish Epidemiology Science Center, Statens Serum Institut, 5 Artillerivej, Copenhagen, DK-2300, Denmark

**Keywords:** Kaposi's sarcoma, non-Hodgkin lymphoma, acquired immunodeficiency syndrome (AIDS), human immunodeficiency virus (HIV), Kaposi's sarcoma herpesvirus (KSHV) also known as human herpesvirus 8

## Abstract

Kaposi's sarcoma (KS), common among persons with acquired immunodeficiency syndrome (AIDS), is caused by KS herpesvirus (KSHV) but whether KSHV causes other malignancies is uncertain. Using linked United States AIDS and cancer registries, we measured the incidence of specific malignancies in persons with AIDS (4–27 months after AIDS onset). We identified associations with KSHV by calculating a relative risk: cancer incidence in persons with KS (all were KSHV-infected) divided by incidence in persons without KS. Using Poisson regression, relative risks were adjusted for human immunodeficiency virus risk group, gender, age, race, and calendar year. We included 189 159 subjects (26 972 with KS). Immunoblastic lymphoma was significantly associated with KS (506 cases; relative risks: unadjusted 2.44, 95%CI 2.00–2.96, adjusted 1.58, 95%CI 1.29–1.93). Only one immunoblastic lymphoma had pleura as primary site. None of 37 other specified malignancies (other non-Hodgkin lymphomas, haematological malignancies, solid tumours) was significantly associated with KS. In summary, the association of immunoblastic lymphoma with KS was specific among examined malignancies and remained significant after statistical adjustment. Our findings, and the previously demonstrated presence of KSHV in the histologically related primary effusion lymphoma, suggest that KSHV is involved in the pathogenesis of some immunoblastic lymphomas. © 2001 Cancer Research Campaign


British Journal of Cancer (2001) 85(9), 1298–1303
© 2001 Cancer Research Campaign
doi: 10.1054/ bjoc.2001.2052, available online at http://www.idealibrary.com on IDEAL

http://www.bjcancer.com

### Cancers associated with Kaposi's sarcoma (KS) in AIDS: a link between KS herpesvirus and immunoblastic lymphoma

**EA Engels[1], PS Rosenberg[2], M Frisch[1,3] and JJ Goedert[1]**

[1]Viral Epidemiology Branch and Biostatistics Branch, Division of Cancer Epidemiology and Genetics, National Cancer Institute, 6120 Executive Blvd., Rockville, MD, USA; [2]Biostatistics Branch, Division of Cancer Epidemiology and Genetics, National Cancer Institute, 6120 Executive Blvd., Rockville MD, USA; [3]Department of Epidemiology Research, Danish Epidemiology Science Center, Statens Serum Institut, 5 Artillerivej, DK-2300 Copenhagen, Denmark

**Summary** Kaposi's sarcoma (KS), common among persons with acquired immunodeficiency syndrome (AIDS), is caused by KS herpesvirus (KSHV) but whether KSHV causes other malignancies is uncertain. Using linked United States AIDS and cancer registries, we measured the incidence of specific malignancies in persons with AIDS (4–27 months after AIDS onset). We identified associations with KSHV by calculating a relative risk: cancer incidence in persons with KS (all were KSHV-infected) divided by incidence in persons without KS. Using Poisson regression, relative risks were adjusted for human immunodeficiency virus risk group, gender, age, race, and calendar year. We included 189 159 subjects (26 972 with KS). Immunoblastic lymphoma was significantly associated with KS (506 cases; relative risks: unadjusted 2.44, 95%CI 2.00–2.96, adjusted 1.58, 95%CI 1.29–1.93). Only one immunoblastic lymphoma had pleura as primary site. None of 37 other specified malignancies (other non-Hodgkin lymphomas, haematological malignancies, solid tumours) was significantly associated with KS. In summary, the association of immunoblastic lymphoma with KS was specific among examined malignancies and remained significant after statistical adjustment. Our findings, and the previously demonstrated presence of KSHV in the histologically related primary effusion lymphoma, suggest that KSHV is involved in the pathogenesis of some immunoblastic lymphomas. © 2001 Cancer Research Campaign

**Keywords**: Kaposi's sarcoma; non-Hodgkin lymphoma; acquired immunodeficiency syndrome (AIDS); human immunodeficiency virus (HIV); Kaposi's sarcoma herpesvirus (KSHV) also known as human herpesvirus 8

Kaposi's sarcoma herpesvirus (KSHV), also known as human herpesvirus 8, is now established as the aetiologic agent of Kaposi's sarcoma (KS) (Antman and Chang, 2000). Most KSHV-infected people are asymptomatic. However, with immunosuppression induced by human immunodeficiency virus type 1 (HIV), risk for KS increases substantially (Martin et al, 1998).

Several lines of evidence suggest that KSHV is also a cause of lymphoma in HIV-infected persons. KSHV is found within circulating B lymphocytes (Blackbourn et al, 1997) and is closely related to Epstein–Barr virus and herpesvirus saimiri, which each cause lymphoma (International Agency for Research on Cancer, 1997, p. 375–494). KSHV might stimulate lymphocytes to proliferate abnormally through virally encoded homologues of cytokines, such as v-interleukin 6, or proteins that interfere with cell cycle control, such as v-cyclin or latent nuclear antigen (Moore et al, 1996; Radkov et al, 2000). KSHV is consistently detected in acquired immunodeficiency syndrome (AIDS)-associated primary effusion lymphoma, a rare tumour arising from pleura, pericardium or peritoneum (Cesarman et al, 1995; Nador et al, 1996; Gessain et al, 1997). The virus is also found in lymphomas occurring in multicentric Castleman's disease (Dupin et al, 2000). Nonetheless, it remains uncertain whether KSHV actually causes these lymphomas or other lymphoma variants among persons with AIDS.

*Received 19 April 2001*
*Revised 13 July 2001*
*Accepted 16 July 2001*

*Correspondence to:* EA Engels

There are few systematic data on the relationship between KSHV and various malignancies, including lymphoma. In the present study, we compared the incidence of various malignancies in persons with AIDS-associated KS (all of whom were KSHV-infected) with the incidence in other persons with AIDS (most of whom were KSHV-uninfected). With this design, a significant association with KS served to identify tumours for which KSHV might be aetiologic. An advantage of our registry-based study was its large size, including over 189 000 persons with AIDS.

### MATERIALS AND METHODS

### Study subjects and matching

We used data from the AIDS-Cancer Match Registry study. As described elsewhere (Frisch et al, 2001), this study provides data on cancer incidence in persons with AIDS, through linkage of cancer and AIDS registries from eleven areas of the United States, namely, the states of Connecticut, Florida, Illinois, Massachusetts, New Jersey, and New York, and the metropolitan areas of Atlanta, Los Angeles, San Diego, San Francisco, and Seattle. Thus, included subjects were those persons with an AIDS diagnosis (as documented in an AIDS registry) occurring in the period of registration covered by the relevant cancer registry, and the occurrence of malignancies in these subjects was identified using the cancer registries. The period of registration varied by cancer registry, but all included 1983–1994, except San Diego, where registration began in 1988, and no data were available after 1996. In the present study, 2 areas were excluded, because the cancer registry included a substantial number of inaccurately diagnosed cases of KS or provided data only on first malignancies.

All subjects were classified according to whether they developed KS at any point during observation, based on diagnoses recorded in either AIDS or cancer registries. For this purpose, we considered cases of KS dated from AIDS onset (under the definition of AIDS, no cases of KS occurred before AIDS) until 60 months after AIDS onset.

### Incidence of malignancies

Using diagnoses recorded in the cancer registries, we calculated the incidence of various malignancies from 4 to 27 months after AIDS onset (post-AIDS period). This 2-year period excluded the 3 months immediately following AIDS (AIDS period), since cancer ascertainment would have been elevated at that time due to heightened diagnostic efforts at AIDS onset. Although we also had data on malignancies arising 28–60 months after AIDS onset, AIDS-related mortality increases steeply during this late period, and we did not have complete survival data. Therefore, to avoid inadvertently including person-time for subjects with unreported deaths (and thus underestimating incidence), we censored follow-up at 27 months after AIDS onset.

Observed malignancies were categorized by site (and, for lymphomas, leukaemias, and melanoma skin cancer, by histology) based on the second edition of International Classification of Diseases for Oncology (ICDO-2) (Percy et al, 1990). Under the Working Formulation (Non-Hodgkin's lymphoma pathologic classification project, 1982), non-Hodgkin lymphomas were classified as low-grade (ICDO-2 codes 9670–9671, 9691–9696); intermediate-grade (9672–9676, 9680–9683, 9697–9698); high-grade, further classified as Burkitt (9687), large cell immunoblastic (9684), or other high-grade (9685–9686); or other/unspecified (all other codes from 9590–9595 and 9670–9714). For calculating post-AIDS incidence of non-Hodgkin lymphomas, we excluded subjects who had non-Hodgkin lymphoma in the AIDS period. Similarly, for cervical cancer, we excluded women diagnosed with cervical cancer in the AIDS period.

### Statistical analysis

For each malignancy, association with KS was assessed by calculating the relative risk, defined as the post-AIDS incidence in subjects with KS divided by the post-AIDS incidence in subjects without KS. We calculated 2-sided 95% confidence intervals for these relative risks using an exact method (Breslow and Day, 1987, pp. 91–95).

For malignancies significantly associated with KS, we used Poisson regression (McCullagh and Nelder, 1989) to adjust the relative risks for gender, age at AIDS (less than 30, 30–39, 40–49, 50+ years), race (white, black, Hispanic, other/unknown), and calendar year (1978–1984, 1985–1988, 1989–1992, 1993–1996). We also controlled for HIV risk group (homosexual male vs. other), because most cases of AIDS-associated KS arise in homosexual males, and this group, compared with other risk groups, may have higher risk for some cancers (e.g. anal cancer) due to factors other than KSHV infection. In an additional analysis, we also adjusted relative risks for CD4 counts at AIDS, which were available for a minority of subjects.

Levels of continuous variables were compared between groups using the 2-sample *t*-test, and the distributions of categorical variables were compared using the $\chi^2$ statistic. Matlab (Version 5, Math Works, Natick, MA) and S-Plus (Version 2000, MathSoft, Seattle, WA) were utilized for calculations. A 5% level of statistical significance was used.

We calculated the attributable risk percent (the proportion of cancer cases, among those with KS, attributable to having KS) as (RR–1)/RR, and the population-attributable risk percent (the proportion of cancer cases, in the entire AIDS population, attributable to having KS) as $p_{KS} \times (RR-1)/[p_{KS} \times (RR-1) + 1]$, where RR was the adjusted relative risk for cancer, given KS, and $p_{KS}$ was the prevalence of KS in the population (Hennekens and Buring, 1987, pp. 87–93). Because KS served to identify persons with KSHV infection, these figures are an estimate of the proportion of cases attributable to KSHV infection.

### RESULTS

### Study subjects

We studied 189 159 subjects: 26 972 (14%) with KS (occurring at any time during follow-up) and 162 187 (86%) without KS (Table 1). The majority (92%) of subjects with KS were homosexual males, and most were white. In contrast, among subjects without KS, only 47% were homosexual males and 57% were black or Hispanic (Table 1). CD4 counts at AIDS diagnosis were available for 67 896 subjects (36%). Among subjects with known CD4 counts, subjects with KS were slightly more immunocompromised than those without KS (mean CD4 counts at AIDS 91 vs. 115 cells $mm^{-3}$, *P* = 0.0001). 68% of subjects with KS had CD4 counts below 100 cells $mm^{-3}$ at AIDS, compared with 55% of subjects without KS. Age did not differ between the 2 groups.

### Relative risks for malignancies

For the post-AIDS period, Table 2 presents relative risks associated with KS, for each of the examined malignancies. Unadjusted relative risks were significantly elevated for several histologic categories of non-Hodgkin lymphoma: intermediate grade lymphomas (relative risk 1.50, 95% confidence interval (CI) 1.26–1.78), high-grade large cell immunoblastic lymphoma (2.44, 95% CI 2.00–2.96), and other/unspecified lymphoma (1.45, 95% CI 1.27–1.66). The increase in large cell immunoblastic lymphoma (IBL) resulted in an elevated relative risk for all types of high-grade lymphoma combined (2.06, 95% CI 1.74–2.45). In analyses adjusted for gender, risk group, age, race, and calendar year, IBL remained associated with KS (adjusted relative risk 1.58, 95% CI 1.29–1.93), but intermediate-grade lymphoma and other/unspecified lymphoma were no longer significantly associated with KS (relative risks 1.05, 95%CI 0.88–1.26, and 1.02, 95%CI 0.89–1.18, respectively). Relative risks for Hodgkin disease, leukaemias, and multiple myeloma were not elevated (Table 2).

For other malignancies (Table 2), only miscellaneous ('other/unknown') tumours were significantly associated with KS (unadjusted relative risk 1.88, 95%CI 1.35–2.59; adjusted relative risk 1.69, 95%CI 1.19–2.39). Among subjects with KS, 34 of these miscellaneous tumours (65%) were classified as 'malignant neoplasm' with unknown primary site, compared with 57 (36%) among other subjects (*P* = 0.0004). Among miscellaneous tumours, tumours were noted at 10 separately specified sites, but none occurred in excess among those with KS (data not shown).

*© 2001 Cancer Research Campaign*

**Table 1** Characteristics of study subjects

| Characteristic | Subjects with Kaposi's sarcoma (*n* = 26 972) | Subjects without Kaposi's sarcoma (*n* = 162 187) | *P* value |
|---|---|---|---|
| Gender and risk group, *n* (%) | | | < 0.0001 |
| Homosexual males | 24 684 (92) | 77 034 (48) | |
| Non-homosexual males | 1842 (7) | 55 769 (34) | |
| Females | 446 (2) | 29 384 (18) | |
| Age at AIDS, mean (sd) | 37 (8) | 37 (10) | 0.54 |
| Race, *n* (%) | | | < 0.0001 |
| White | 18 796 (70) | 66 916 (41) | |
| Black | 3581 (13) | 57 387 (35) | |
| Hispanic | 4179 (15) | 35 831 (22) | |
| Other/unknown | 416 (2) | 2053 (1) | |
| CD4 count at AIDS (cells/mm$^3$), *n* (%)[a] | | | 0.0001 |
| 0–24 | 2605 (35) | 16 600 (28) | |
| 25–49 | 1142 (15) | 7116 (12) | |
| 50–99 | 1409 (19) | 9757 (16) | |
| 100–149 | 880 (12) | 8449 (14) | |
| 150+ | 1495 (20) | 18 443 (31) | |
| Mean CD4 count (SD) | 91 (125) | 115 (144) | |

[a]For CD4 count at AIDS, 19 441 subjects (72%) with Kaposi's sarcoma and 101 822 subjects (63%) without Kaposi's sarcoma had missing values.

### Large cell immunoblastic lymphoma (IBL)

There were 506 IBLs, with 151 occurring in individuals with KS (incidence 0.42%/year) and 355 occurring in individuals without KS (0.17%/year). As noted, the association between KS and IBL was significant even after adjustment for gender, risk group, age, race, and calendar year (adjusted relative risk 1.58, 95%CI 1.29–1.93). In an analysis restricted to subjects with CD4 counts at AIDS, further adjustment for CD4 count did not affect the relative risk (1.50, 1.07–2.10).

The strength of association between KS and IBL did not differ significantly across strata defined by gender and risk group, race, or CD4 count at AIDS (Table 3), or during different calendar periods (data not shown). However, there was some evidence that the strength of association varied across age groups ($P$ = 0.06, Table 3). Among subjects less than 30 years old at AIDS, the relative risk of IBL associated with KS was 1.19 (95%CI 0.63–2.26), which was significantly less than the relative risk among older subjects (2.68, 95%CI 2.19–3.28; $P$ = 0.01 for difference in relative risks). Even after adjustment for gender, risk group, race, and calendar year, relative risk of IBL associated with KS was lower for subjects younger than 30 years ($P$ = 0.01).

Primary sites of IBLs among subjects with KS were: lymph node (81 cases, 54%), brain (33 cases, 22%), and other sites (37 cases, 25%). Sites of IBLs among the other subjects were similar: lymph node (192 cases, 54%), brain (64 cases, 18%), and other sites (99 cases, 28%). Notably, among persons with KS, only one IBL (0.7%) was identifiable as a primary effusion lymphoma, with pleura as the primary site. 3 subjects without KS had IBLs arising in the heart. There were no IBLs with peritoneum as primary site.

Immunophenotype was available for 178 IBLs (35%). Immunophenotyping became more common over time, with data available for 0 of 8 IBLs occurring in 1978–1984, 23 of 99 (23%) occurring in 1985–1988, and 155 of 399 (39%) occurring in 1989–1996. For IBLs with immunophenotyping, 168 (94%) were B cell, 9 (5%) were T cell, and one (1%) had neither B nor T cell markers. The occurrence of B cell IBL was significantly associated with a diagnosis of KS (unadjusted relative risk 2.64, 95%CI 1.87–3.69; adjusted relative risk 1.90, 1.35–2.68). T-cell IBL was also associated with KS, although not significantly (unadjusted relative risk 2.87, 0.46–13.44; adjusted relative risk 2.25, 0.52–9.76).

Using the adjusted KS-associated relative risk of 1.58 for IBL, we calculated the proportion of IBL cases attributable to KSHV, assuming that infection with this virus explained the association with KS and that the association was causal. Among persons with KS, 37% of IBLs were attributable to KSHV (attributable risk percent, 37%). Among all persons with AIDS, 8% of IBLs were attributable to KSHV (population attributable risk percent, 8%).

### DISCUSSION

The most important finding in this study was the demonstration of a relationship between large cell immunoblastic lymphoma (IBL) and KS. Unique among the 37 specified types of malignancy that we examined, IBL was the only tumour significantly associated with KS both in unadjusted analyses and in analyses adjusted for possible confounding factors, including HIV risk group and CD4 count. The association between KS and IBL was not likely due to primary effusion lymphoma occurring among our subjects. Primary effusion lymphomas have an immunoblastic histology (Nador et al, 1996), but among our study subjects with KS, only one IBL arose in a serous body cavity (pleura).

Our findings prompt consideration of the possibility that KSHV is aetiologically involved in some AIDS-associated IBLs. As a consequence of our study design, the excess of IBLs that we observed occurred in persons who also had KS. Thus, our results may indicate that specific defects in immune control of KSHV predispose some immunosuppressed individuals to develop either or both of these tumours. On the basis of the magnitude of association between KS and IBL, we estimated that KSHV may be present and etiologically important in 37% of IBLs arising in persons with KS (attributable risk percent 37%). Notably, the estimates of relative risk and attributable risk may have been too low, because some subjects in the referent group (those without KS)

 *© 2001 Cancer Research Campaign*

**Table 2** Relative risks for malignancies in association with Kaposi's sarcoma

| Site, histology | $n_{KS}$ | $n_{no\ KS}$ | Relative risk (exact 95% CI)[a] |
|---|---|---|---|
| Non-Hodgkin lymphoma | 632 | 2,260 | *1.60* (1.47–1.75) |
| Low-grade | 5 | 22 | 1.30 (0.39–3.53) |
| Intermediate-grade | 169 | 648 | *1.50* (1.26–1.78) |
| High-grade | 186 | 517 | *2.06* (1.74–2.45) |
| Burkitt | 16 | 70 | 1.31 (0.71–2.28) |
| Immunoblastic | 151 | 355 | *2.44* (2.00–2.96) |
| Other high-grade | 19 | 92 | 1.19 (0.68–1.96) |
| Other/unspecified | 272 | 1,073 | *1.45* (1.27–1.66) |
| Leukaemia | 3 | 24 | 0.72 (0.14–2.37) |
| Acute myeloid | 0 | 10 | 0 (0–2.01) |
| Acute lymphoblastic | 1 | 1 | 5.76 (0.07–452) |
| Chronic myeloid | 0 | 1 | 0 (0–109) |
| Chronic lymphocytic | 0 | 1 | 0 (0–109) |
| Other/unspecified | 2 | 11 | 1.05 (0.11–4.80) |
| Hodgkin disease | 8 | 59 | 0.78 (0.32–1.64) |
| Multiple myeloma | 3 | 10 | 1.73 (0.31–6.71) |
| Oral cavity and pharynx | 7 | 42 | 0.96 (0.36–2.16) |
| Oesophagus | 2 | 8 | 1.44 (0.15–7.21) |
| Stomach | 3 | 16 | 1.08 (0.20–3.77) |
| Small intestine | 0 | 2 | 0 (0–20.0) |
| Colon | 5 | 15 | 1.92 (0.55–5.55) |
| Rectum | 2 | 19 | 0.61 (0.07–2.51) |
| Anus | 5 | 33 | 0.87 (0.27–2.25) |
| Liver | 2 | 19 | 0.61 (0.07–2.51) |
| Pancreas | 0 | 11 | 0 (0–1.80) |
| Upper respiratory sites | 2 | 19 | 0.61 (0.07–2.51) |
| Lung | 22 | 162 | 0.78 (0.48–1.22) |
| Kidney | 0 | 15 | 0 (0–1.27) |
| Bladder | 1 | 7 | 0.82 (0.02–6.40) |
| Breast | 0 | 9 | 0 (0–27.2) |
| Ovary | 0 | 1 | 0 (0–1310) |
| Uterus | 0 | 4 | 0 (0–76.9) |
| Cervix | 2 | 37 | 3.71 (0.43–14.4) |
| Vulva and vagina | 0 | 8 | 0 (0–31.3) |
| Testis | 6 | 16 | 1.79 (0.57–4.82) |
| Prostate | 6 | 22 | 1.30 (0.43–3.32) |
| Penis | 0 | 5 | 0 (0–3.92) |
| Thyroid | 3 | 3 | 5.76 (0.77–43.0) |
| Central nervous system | 7 | 40 | 1.01 (0.38–2.27) |
| Melanoma | 3 | 19 | 0.91 (0.17–3.09) |
| Musculoskeletal sites | 5 | 9 | 3.20 (0.84–10.6) |
| Other/unknown | 52 | 159 | *1.88* (1.35–2.59) |

Abbreviations: $n_{KS}$ and $n_{no\ KS}$, number of malignancies occurring in subjects with or without Kaposi's sarcoma, respectively; CI, confidence interval. [a]Relative risks that are in italic are statistically significant ($P < 0.05$). All confidence intervals are two-sided 95% intervals, except when the observed relative risk is zero, in which case the one-sided 95% confidence interval is shown.

were also KSHV-infected. Nonetheless, it seems likely that the pathogenesis of IBL is complex and multifactorial. Epstein–Barr virus is found in the majority of IBLs and primary effusion lymphomas (Shibata et al, 1993; Nador et al, 1996), and it is possible that this virus acts in concert with KSHV to cause IBL.

Laboratory data, though scant, lend support to our epidemiological findings. In their original description of KSHV in primary effusion lymphoma, Cesarman et al did not find KSHV in any of 8 other AIDS-associated lymphomas characterized as 'immunoblastic plasmacytoid' in histology (Cesarman et al, 1995). Other investigators subsequently found KSHV in 17–22% of IBLs arising outside the pleura (Otsuki et al, 1996; Gessain et al, 1997; Boye Hansen et al, 2000). The level of KSHV in positive cases was low, raising the possibility that the virus was present only in normal circulating B lymphocytes within these tumours. However, in a recent preliminary report, Cesarman and Knowles described the presence of KSHV in high copy number in tumour cells from 4 additional AIDS lymphomas, all of which were non-effusion IBLs (Cesarman and Knowles, 1999). Although these studies were small, overall they are consistent with KSHV being present and playing a role in a minority of IBL cases arising in AIDS, and are in accord with our estimate of population attributable risk (8%). Other histologic types of lymphoma (e.g. Burkitt or Burkitt-like tumours) have consistently been KSHV-negative in laboratory studies (Cesarman et al, 1995; Otsuki et al, 1996; Gessain et al, 1997).

Additional, conflicting data on the relationship between KSHV and AIDS-associated non-Hodgkin lymphoma are provided by 2 other studies. Using clinical records and autopsy material, Ridolfo and colleagues identified 60 cases of non-Hodgkin lymphoma in 363 persons dying with AIDS (Ridolfo et al, 1996). 16 of these lymphomas were found in persons with KS, of which 13 (81%) were 'large-cell immunoblastic plasmacytoid' in histology. Notably, individuals whose AIDS index diagnosis was KS were significantly more likely to develop non-Hodgkin lymphoma than those with the more common index diagnosis of *Pneumocystis carinii* pneumonia (relative risk 5.3). On the other hand, Gérard et al observed no difference in KSHV seroprevalence between 63 AIDS lymphoma cases and 126 HIV-infected controls (Gerard et al, 2001). A limitation of the Gérard study, however, was that cases and controls were matched for history of KS, which might have eliminated some differences in KSHV prevalence. Also, serological assays for KSHV can misclassify infection status (Engels et al, 2000).

Lymphomas arising in multicentric Castleman's disease could partially explain the relationship between KS and IBL. Among HIV-infected persons, KS is associated with the plasmablastic variant of multicentric Castleman's disease (Soulier et al, 1995; Dupin et al, 2000). In multicentric Castleman's disease, KSHV is found in plasmablasts in the mantle zone of involved lymph nodes and in secondary plasmablastic lymphomas (Dupin et al, 2000). Plasmablastic lymphomas closely resemble IBL histologically (Dupin et al, 2000; Anagnostopoulos et al, 2001). Indeed, the ICDO-2 lacks a code for plasmablastic lymphoma, and plasmablastic lymphoma cases might have been classified as IBLs in our study. Nonetheless, multicentric Castleman's disease is rare (Oksenhendler et al, 1996) and is unlikely to account entirely for our findings.

In our study, the finding of an association between IBL and KS was given strength by its consistency across strata defined by HIV risk group, gender, race and CD4 count. This consistency argues

© 2001 Cancer Research Campaign 

**Table 3** Stratum-specific relative risks for large cell immunoblastic lymphoma, in association with Kaposi's sarcoma

| Stratum | Subjects[a] | IBLs, n | Relative risk associated with Kaposi's sarcoma (95% CI) | *P*-value for difference in relative risk across strata |
|---|---|---|---|---|
| Gender and risk group | | | | 0.84 |
| Homosexual males | 99 357 | 414 | 1.62 (1.32–1.98) | |
| Non-homosexual males | 56 867 | 62 | 2.10 (0.76–5.78) | |
| Females | 29 574 | 30 | 2.40 (0.33–17.61) | |
| Age, y | | | | 0.06 |
| < 30 | 32 617 | 71 | 1.19 (0.63–2.26) | |
| 30–39 | 87 212 | 216 | 2.85 (2.16–3.77) | |
| 40–49 | 49 067 | 174 | 2.67 (1.93–3.68) | |
| 50+ | 16 902 | 45 | 1.95 (0.97–3.94) | |
| Race | | | | 0.14 |
| White | 83 517 | 358 | 1.77 (1.42–2.20) | |
| Black | 60 402 | 51 | 3.90 (1.96–7.80) | |
| Hispanic | 39 440 | 88 | 1.58 (0.89–2.79) | |
| Other/unknown | 2439 | 9 | 3.96 (1.06–14.76) | |
| CD4 count at AIDS, cells/mm$^3$ | | | | 0.14 |
| <25 | 18 995 | 60 | 1.49 (0.79–2.80) | |
| 25–49 | 8154 | 33 | 2.56 (1.22–5.37) | |
| 50–99 | 11 007 | 45 | 3.19 (1.72–5.93) | |
| 100–149 | 9210 | 26 | 0.72 (0.17–3.04) | |
| 150+ | 19 667 | 36 | 3.11 (1.42–6.82) | |

Abbreviations: IBL = large cell immunoblastic lymphoma; CI = confidence interval. [a]These analyses for risk in the post-AIDS period were restricted to the 185 798 subjects who did not have a previous (AIDS period) diagnosis of non-Hodgkin lymphoma. Additionally, for relative risks within strata defined by CD4 count at AIDS, analyses were restricted to the 67 033 (36%) of these subjects who had CD4 counts reported.

against the possibility that unknown factors other than KSHV infection explain the observed association between KS and IBL. Interestingly, this association was strongest in persons at least 30 years old at AIDS onset. KSHV-related risk for IBL may be higher in older individuals due to loss of control of KSHV infection with aging, accumulation of somatic genetic errors, or acquisition of additional cofactors for lymphomagenesis. Alternatively, the association between KS and IBL could have been diluted in younger subjects if clinically inapparent KSHV infection was especially common in that subgroup. Arguing against this second explanation, limited data from cohort studies of homosexual men do not suggest markedly higher KSHV prevalence among younger men (e.g., those born after 1955) (Martin et al, 1998; Melbye et al, 1998).

As noted, the association between KS and IBL was specific. After we adjusted for possible confounders, no other type of lymphoma was related to KS. Risk was increased for miscellaneous tumours, but many of these had non-specific histological codes and may have been misclassified cases of KS. Risk for myleoma was not elevated, which argues against a role for KSHV in this disease despite the controversial identification of KSHV in cultured bone marrow stromal cells from myeloma patients (Berenson and Vescio, 1999; Tarte et al, 1999). Similarly, prostate cancer risk was not increased, even though some epidemiological evidence implicates a sexually transmitted agent (Hayes et al, 2000), and KSHV has been variably found in prostate tissue (Monini et al, 1996; Staskus et al, 1997) and semen (Monini et al, 1996; Howard et al, 1997; Pauk et al, 2000).

Our study complements several previous systematic studies of cancer in KSHV infection. In a survey of South African cancer patients, Sitas et al did not find elevated KSHV-seroprevalence associated with cancers other than KS (Sitas et al, 1999). A potential limitation of that study, however, was its reliance on antibody testing to diagnose KSHV infection (Engels et al, 2000). Using a design similar to that of our study, Biggar et al did not find an excess incidence of malignancies among HIV-uninfected persons with KS (Biggar et al, 1994). On the other hand, among HIV-uninfected Israelis with KS, Iscovich and colleagues noted a 4-fold excess of non-Hodgkin lymphoma and no other elevations in cancer risk except for melanoma (Iscovich et al, 1999). Both investigations provided no data on HIV-infected persons and were hampered by their relatively small size, which precluded detection of increases in rare malignancies.

Despite our study's large size, we were limited in our ability to identify a potential association for some rare malignancies (e.g., leukaemias). The study was also underpowered to detect associations for female-specific cancers because we had few women with KS. However, there is no other evidence of a casual link between these malignancies and KSHV.

In summary, our study of individuals with AIDS demonstrated an increased risk for IBL among persons with KS. This association with KS was specific among malignancies, remained statistically significant after adjustment for possible confounding factors, and was consistent across subgroups defined by gender, HIV risk group and race. Given the paucity of laboratory data on KSHV in IBL, additional laboratory studies, particularly of IBLs arising in persons with KS, are needed to determine whether KSHV plays an aetiologic role. Further epidemiological studies will also be informative.

### ACKNOWLEDGEMENTS

The National AIDS-Cancer Match Registry study used data from AIDS registries established by the Centers for Disease Control and Prevention and from state and local cancer registries, some of which were supported by the National Cancer Institute's Surveillance, Epidemiology, and End Results Program.

 *© 2001 Cancer Research Campaign*

Contributors of registry data to this study (AIDS registry, cancer registry) were: Awal Kahn, Nancy Stroup, Atlanta; Ken Carley, John Flannery, Connecticut; Lisa Conti, Edward Trapido, Florida; Chet Kelly, Holly L. Howe, Illinois; Amy Rock Wohl, Dennis Deapen, Los Angeles; Margaret Owen, Susan Gershman, Massachusetts; Sam Costa, Betsy Kohler, New Jersey; James Fordyce, Maria J. Schymura, New York City; Brian Gallagher, Maria J. Schymura, New York State; Michelle Ginsberg, Hoda Anton-Culver, San Diego; Ling Chin Hsu, Dee West, San Francisco; and Sharon Hopkins, Charles Wiggins, Seattle. The registry contributions are attributed to these specific contributors, but we are also indebted to the many individuals who developed and maintained these highly complex systems and to those who contributed primary data to them.

The authors also acknowledge the contributions of Tim Borges, Bob Stafford and PY Lu, at Oak Ridge National Laboratories (Tennessee), for implementation of the registry match. We thank Kathryn Schleeter, Tim McNeel and Steve Scoppa, at Information Management Systems (Silver Spring, Maryland) for data management. The research described herein was approved by ethical committees at the National Cancer Institute and participating AIDS and cancer registries.

### REFERENCES

Anagnostopoulos I, Dallenbach F and Stein H (2001) Diffuse large cell lymphomas. In: *Neoplastic hematopathology*, Knowles DM (ed) pp 855–913. Lippincott Williams and Wilkins: Philadelphia

Antman K and Chang Y (2000) Kaposi's sarcoma. *N Engl J Med* **342**: 1027–1038

Berenson JR and Vescio RA (1999) HHV-8 is present in multiple myeloma patients. *Blood* **93**: 3157–3159

Biggar RJ, Curtis RE, Cote TR, Rabkin CS and Melbye M (1994) Risk of other cancers following Kaposi's sarcoma: relation to acquired immunodeficiency syndrome. *Am J Epidemiol* **139**: 362–368

Blackbourn DJ, Ambroziak J, Lennette E, Adams M, Ramachandran B and Levy JA (1997) Infectious human herpesvirus 8 in a healthy North American blood donor. *Lancet* **349**: 609–611

Boye Hansen P, Penkowa M, Kirk O, Skinhoj P, Pedersen C, Lisse I, Kiss K, Zhou Z-G and Hamiton-Dutoit SJ (2000) Human immunodeficiency virus-associated malignant lymphoma in eastern Denmark diagnosed from 1990 to 1996: clinical features, histopathology, and association with Epstein-Barr virus and human herpesvirus-8. *Eur J Haematol* **64**: 368–375

Breslow NE and Day NE (1987) *Statistical methods in cancer research, volume 2* IARC: Lyon

Cesarman E, Chang Y, Moore PS, Said JW and Knowles DM (1995) Kaposi's sarcoma-associated herpesvirus-like DNA sequences in AIDS-related body-cavity-based lymphomas. *N Engl J Med* **332**: 1186–1191

Cesarman E and Knowles DM (1999) The role of Kaposi's sarcoma-associated herpesvirus (KSHV/HHV-8) in lymphoproliferative disease. *Sem Cancer Biol* **9**: 165–174

Dupin N, Diss TL, Kellam P, Tulliez M, Du M-Q, Sicard D, Weiss RA, Isaacson PG and Boshoff C (2000) HHV-8 is associated with a plasmablastic variant of Castleman disease that is linked to HHV-8-positive plasmablastic lymphoma. *Blood* **95**: 1406–1412

Engels EA, Whitby D, Goebel PB, Stossel A, Waters D, Pintus A, Contu L, Biggar RJ and Goedert JJ (2000) Identifying human herpesvirus 8 infection: performance characteristics of serological assays. *J Acquir Immune Defic Syndr* **23**: 346–354

Frisch M, Biggar RJ, Engels EA and Goedert JJ (2001) Association of cancer with AIDS-related immunosuppression in adults. *JAMA* **285**: 1736–1745

Gérard L, Agbalika F, Sheldon J, Maillard A, Schulz TF and Oksenhendler E (2001) No increased human herpesvirus 8 seroprevalence in patients with HIV-associated non-Hodgkin's lymphoma. *J Acquir Immune Defic Syndr* **26**: 182–184

Gessain A, Briere J, Angelin-Duclos C, Valensi F, Merle Beral H, Davi F, Nicola M-A, Sudaka A, Fouchard N, Gabarre J, Troussard X, Dulmet E, Audouin J, Diebold J and de The G (1997) Human herpes virus 8 (Kaposi's sarcoma herpes virus) and malignant lymphoproliferations in France: a molecular study of 250 cases including two AIDS-associated body cavity based lymphomas. *Leukemia* **11**: 266–272

Hayes RB, Pottern LM, Strickler H, Rabkin C, Pope V, Swanson GM, Greenberg RS, Schoenberg JB, Liff J, Schwartz AG, Hoover RN and Fraumeni J.F,Jr. (2000) Sexual behaviour, STDs and risk for prostate cancer. *Br J Cancer* **82**: 718–725

Hennekens CH and Buring JE (1987) *Epidemiology in medicine.* Little, Brown: Boston

Howard MR, Whitby D, Bahadur G, Suggett F, Boshoff C, Tenant-Flowers M, Schulz TF, Kirk S, Matthews S, Weller IVD, Tedder RS and Weiss RA (1997) Detection of human herpesvirus 8 DNA in semen from HIV-infected individuals but not from healthy semen donors. *AIDS* **11**: F15–F19

International Agency for Research on Cancer (1997) *Epstein-Barr virus and Kaposi's sarcoma herpesvirus/human herpesvirus 8.* Lyon, France

Iscovich J, Boffetta P and Brennan P (1999) Classic Kaposi's sarcoma as a first primary neoplasm. *Int J Cancer* **80**: 173–177

Martin JN, Ganem DE, Osmond DH, Page-Shafer KA, Macrae D and Kedes DH (1998) Sexual transmission and the natural history of human herpesvirus 8 infection. *N Engl J Med* **338**: 948–954

McCullagh P and Nelder JA (1989) *Generalized linear models*. Chapman and Hall: London

Melbye M, Cook PM, Hjalgrim H, Begtrup K, Simpson GR, Biggar RJ, Ebbesen P and Schulz TF (1998) Risk factors for Kaposi's-sarcoma-associated herpesvirus (KSHV/HHV-8) seropositivity in a cohort of homosexual men, 1981–1996. *Int J Cancer* **77**: 543–548

Monini P, de Lellis L, Fabris M, Rigolin F and Cassai E (1996) Kaposi's sarcoma-associated herpesvirus DNA sequences in prostate tissue and human semen. *N Engl J Med* **334**: 1168–1172

Moore PS, Boshoff C, Weiss RA and Chang Y (1996) Molecular mimicry of human cytokine and cytokine response pathway genes by KSHV. *Science* **274**: 1739–1744

Nador RG, Cesarman E, Chadburn A, Dawson DB, Ansari MQ, Said J and Knowles DM (1996) Primary effusion lymphoma: a distinct clinicopathologic entity associated with the Kaposi's sarcoma-associated herpes virus. *Blood* **88**: 645–656

Non-Hodgkin's lymphoma pathologic classification project (1982) National Cancer Institute sponsored study of classifications of non-Hodgkin's lymphoma. Summary and description of a working formulation for clinical usage. *Cancer* **49**: 2112–2135

Oksenhendler E, Duarte M, Soulier J, Cacoub P, Welker Y, Cadranel J, Cazals-Hatem D, Autran B, Clauvel J and Raphael M (1996) Multicentric Castleman's disease in HIV infection: a clinical and pathological study of 20 patients. *AIDS* **10**: 61–67

Otsuki T, Kumar S, Ensoli B, Kingma DW, Yano T, Stetler-Stevenson M, Jaffe ES and Raffeld M (1996) Detection of HHV-8/KSHV DNA sequences in AIDS-associated extranodal lymphoid malignancies. *Leukemia* **10**: 1358–1362

Pauk J, Huang M-L, Brodie SJ, Wald A, Koelle DM, Schacker T, Celum C, Selke S and Corey L (2000) Mucosal shedding of human herpesvirus 8 in men. *N Engl J Med* **343**: 1369–1377

Percy C, Van Holten V and Muir C (1990) *International classification of diseases for oncology*. World Health Organization: Geneva

Radkov SA, Kellam P and Boshoff C (2000) The latent nuclear antigen of KSHV targets the retinoblastoma/E2F pathway and with H-ras transforms primary rat cells. *Nature Med* **6**: 1121–1127

Ridolfo AL, Santambrogio S, Mainini F, Vago L, Gervasoni C, Gori A, Parravicini C, d'Arminio Monforte A and Galli M (1996) High frequency of non-Hodgkin's lymphoma in patients with HIV-associated Kaposi's sarcoma. *AIDS* **10**: 181–185

Shibata D, Weiss LM, Hernandez AM, Nathwani BN, Bernstein L and Levine AM (1993) Epstein-Barr virus-associated non-Hodgkin's lymphoma in patients infected with the human immunodeficiency virus. *Blood* **81**: 2102–2109

Sitas F, Carrara H, Beral V, Newton R, Reeves G, Bull D, Jentsch U, Pacella-Norman R, Bourboulia D, Whitby D, Boshoff C and Weiss R (1999) Antibodies against human herpesvirus 8 in black South African patients with cancer. *N Engl J Med* **340**: 1863–1871

Soulier J, Grollet L, Oksenhendler E, Cacoub P, Cazals-Hatem D, Babinet P, d'Agay M, Clauvel J, Raphael M, Degos L and Sigaux F (1995) Kaposi's sarcoma-associated herpesvirus-like DNA sequences in multicentric Castleman's disease. *Blood* **86**: 1276–1280

Staskus KA, Zhong W, Gebhard K, Herndier B, Wang H, Renne R, Beneke J, Pudney J, Anderson DJ, Ganem D and Haase AT (1997) Kaposi's sarcoma-associated herpesvirus gene expression in endothelial (spindle) tumor cells. *J Virol* **71**: 715–719

Tarte K, Chang Y and Klein B (1999) Kaposi's sarcoma-associated herpesvirus and multiple myeloma: lack of criteria for causality. *Blood* **93**: 3159–3163

© 2001 Cancer Research Campaign
